# Anatomical evidence for scent guided foraging in the turkey vulture

**DOI:** 10.1038/s41598-017-17794-0

**Published:** 2017-12-12

**Authors:** Nathan P. Grigg, Justin M. Krilow, Cristian Gutierrez-Ibanez, Douglas R. Wylie, Gary R. Graves, Andrew N. Iwaniuk

**Affiliations:** 1 Department of Neuroscience, University of Lethbridge, Lethbridge, AB Canada; 2 Neuroscience and Mental Health Institute, University of Alberta, Edmonton, AB Canada; 3 Department of Vertebrate Zoology, National Museum of Natural History, Smithsonian Institution, Washington, DC USA

## Abstract

The turkey vulture (*Cathartes aura*) is a widespread, scavenging species in the Western Hemisphere that locates carrion by smell. Scent guided foraging is associated with an expansion of the olfactory bulbs of the brain in vertebrates, but no such neuroanatomical data exists for vultures. We provide the first measurements of turkey vulture brains, including the size of their olfactory bulbs and numbers of mitral cells, which provide the primary output of the olfactory bulbs. Comparative analyses show that the turkey vulture has olfactory bulbs that are 4× larger and contain twice as many mitral cells as those of the sympatric black vulture (*Coragyps atratus*), despite having brains that are 20% smaller. The turkey vulture has the largest olfactory bulbs in absolute terms and adjusted for brain size among birds, but the number of mitral cells is proportional to the size of their olfactory bulbs. The combination of large olfactory bulbs, high mitral cell counts and a greatly enlarged nasal cavity likely reflects a highly sensitive olfactory system. We suggest that this sensitive sense of smell allowed the turkey vulture to colonize biomes that are suboptimal for scavenging birds and become the most widespread vulture species in the world.

## Introduction

The overall structure of the olfactory system in vertebrates is highly conserved^1^. This generality even extends to the relatively small olfactory bulbs of birds^2,3^, which were originally considered to be microsmatic or anosmic^4^. Overwhelming evidence has accumulated since the 1960s showing that olfaction plays an important role in finding food^5–8^, navigation^6,9–11^, mate selection^12^ and individual and species recognition in birds^13–16^. Olfaction is now recognized as an important sensory modality in birds, but its relative importance varies greatly among species and this is indicated by olfactory bulb size. Following the principle of proper mass^17^, olfactory bulb size reflects processing capacity such that species with large olfactory bulbs have greater olfactory sensitivity and/or acuity than species with small olfactory bulbs. The relationship between olfactory bulb size and sensory abilities is supported by several anatomical studies^2,3,18^. For example, the enlargement of the olfactory bulbs in the kiwi (*Apteryx australis*)^19^ is reflected in their superior performance in behavioral experiments^20^ and further emphasized the importance of olfaction in this species. In a more recent study, Corfield *et al*.^3^ found substantial variation in relative olfactory bulb size across 135 bird species that was related to foraging behavior and habitat, reinforcing the relationship between sense of smell and olfactory bulb size across species.

One group that has remained relatively under studied^21,22^ in terms of olfaction is the New World Vultures (Cathartidae)^23^. Both Old World and New World vultures feed almost exclusively on carrion^22^, but evolved independently of each other^23–25^. Old World vultures appear to rely exclusively on visual cues to find carcasses whereas species within the Cathartidae vary in the degree to which they use olfaction^8,26^. Based on field observations and experiments, the turkey vulture (*Cathartes aura*), the New World vulture with the largest geographic range, relies almost entirely on olfaction to find carrion in dense forests and they are attracted to the scent of carrion in the absence of visual cues^8,26,27^. In stark contrast, the largely sympatric black vulture (*Coragyps atratus*) is apparently not attracted to the scent of carrion and in the absence of visual cues does not approach carrion^8^. Black vultures also tend to soar at greater heights, feed on larger carcasses, often displace turkey vultures at carcasses, and are thought by some authors to track feeding aggregations of turkey vultures to locate carcasses^8,28^.

Turkey vultures have large and complexly folded nasal sinuses, indicating a relatively large olfactory epithelial surface area compared with other vultures and appear to have enlarged olfactory bulbs, based on linear measurements of endocasts^2,8^. Although these data provide preliminary evidence that turkey vultures have enlarged olfactory bulbs, endocast data do not accurately reflect olfactory bulb size^3,19^ and direct neuroanatomical measurements are lacking for any brain regions in vultures. In addition, there are no published comparative data on the number of mitral cells in avian olfactory bulbs. Mitral cells are critical in the processing of olfactory information because they receive input from olfactory receptors and send output to other regions of the brain, where it can be perceived and integrated with other sensory information^1,29–31^. Here, we investigate olfactory bulb size and mitral cell counts in turkey and black vultures and compare them with those of other bird species.

## Results

### Comparison of turkey and black vultures

**Figure 1:**
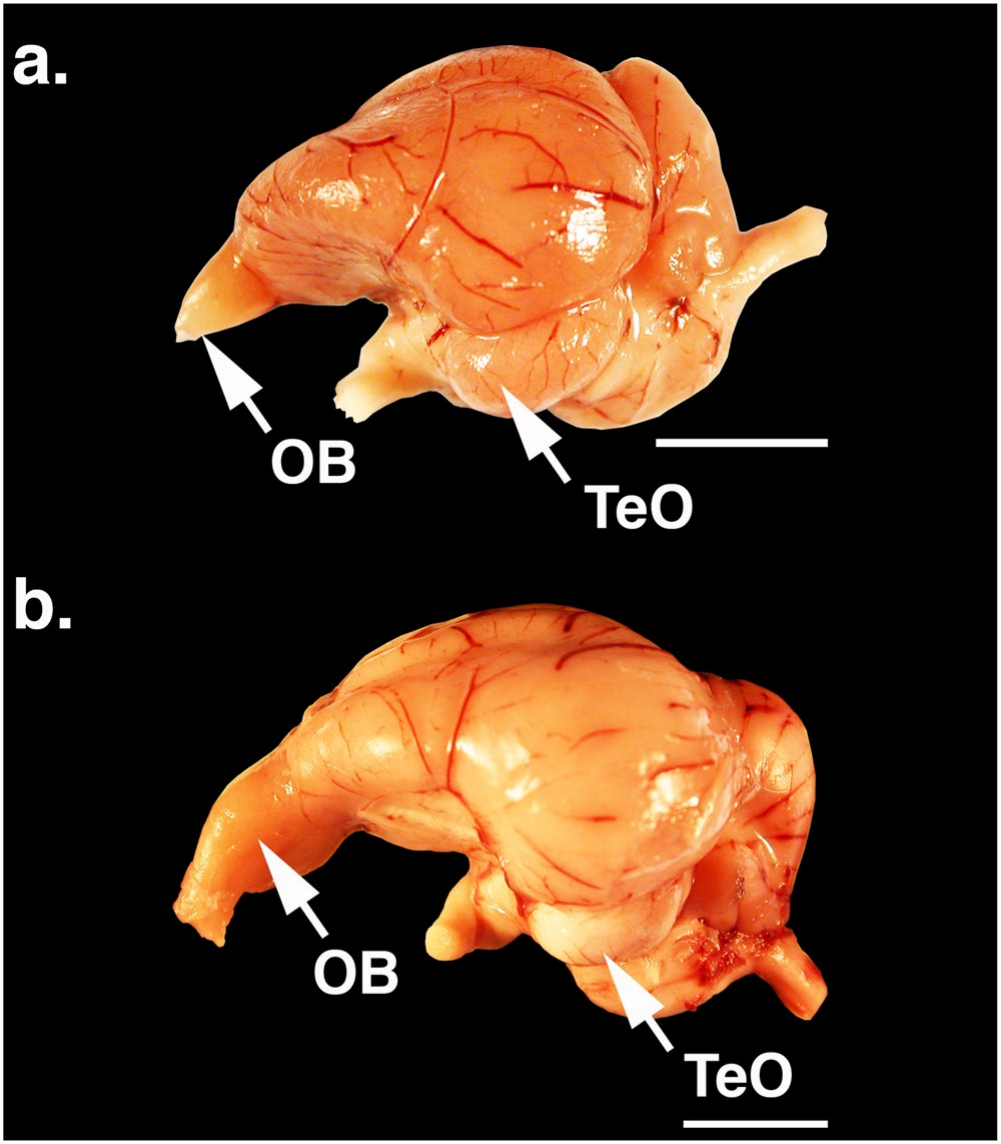
Photos of the brains of: (**a**) black vulture (*Coragyps atratus*); and (**b**) turkey vulture (*Cathartes aura*). The arrows indicate the olfactory bulbs (OB) and optic lobes (TeO). Scale bars = 10 mm.

Differences in morphology were readily apparent in the brains of the two species (Fig. 1); the turkey vulture has significantly larger olfactory bulbs than the black vulture (Table 1). On average, the olfactory bulbs of turkey vultures are 4× larger with twice as many mitral cells as those of black vultures, despite having brains that are 20% smaller. The black vulture has a significantly larger telencephalon and entopallium than the turkey vulture (Table 1), but this is associated with the overall larger size of black vulture brains.

**Table 1:** Average brain region volumes (±standard deviations) and numbers of mitral cells in turkey vultures and black vultures and associated p-values of two sample t-tests of absolute volumes and numbers are shown. The museum catalog numbers of the specimens examined are: USNM647470, USNM647480, USNM647449, USNM647485, USNM647441, USNM647440.

Measurement	turkey vulture (*Cathartes aura*) (n = 3)	black vulture (*Coragyps atratus*) (n = 3)	p
Whole brain volume (mm^3^)	9,211.97 ± 203.56	11,579.22 ± 753.16	**0**.**006**
Telencephalon volume (mm^3^)	6,154.25 ± 81.16	8,113.36 ± 626.07	**0**.**006**
Optic tectum volume (mm^3^)	222.96 ± 20.17	219.09 ± 12.63	0.79
Nucleus rotundus volume (mm^3^)	13.98 ± 1.92	15.79 ± 0.31	0.21
Entopallium volume (mm^3^)	100.25 ± 9.90	129.11 ± 7.10	**0**.**01**
Olfactory bulb volume (mm^3^)	224.87 ± 34.92	54.28 ± 7.04	**0**.**001**
Number of mitral cells	162,565 ± 16,094	86,550 ± 10,154	**0**.**002**

**Figure 2:**
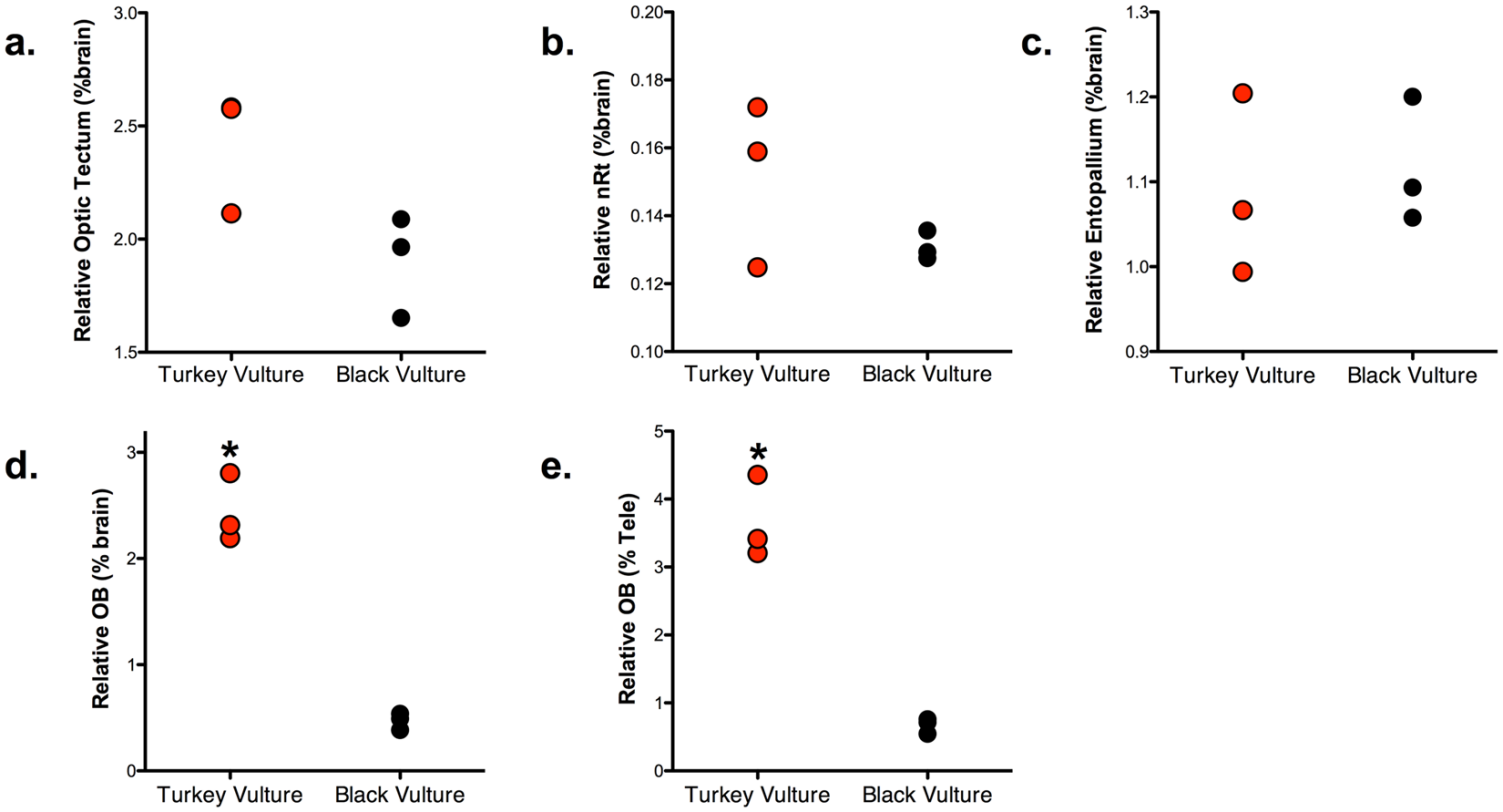
Univariate scatterplots of the relative size of the following brain regions in turkey vultures (red) and black vultures (black), expressed as a proportion of total brain volume: (**a**) optic tectum; (**b**) nucleus rotundus (nRt); (**c**) entopallium; and (**d**) olfactory bulbs (OB). (**e**) A univariate scatterplot of olfactory bulb volume expressed as a percentage of total telencephalon volume. Significant differences between the two species are indicated by an asterisk.

Because the two species differ significantly in absolute brain mass and telencephalon volume, we divided the size of each brain region by overall brain size to compare relative brain region sizes. No significant differences were detected between the two species in the proportional sizes of the optic tectum (p = 0.06), nucleus rotundus (p = 0.21) or entopallium (p = 0.72, Fig. 2a–c). Thus, there are no significant differences in the relative size of brain regions within the tectofugal visual pathway.

However, the difference in olfactory bulb size was striking. Relative to brain size, the turkey vulture has olfactory bulbs that are 4× larger than that of the black vulture (p = 0.0005, Fig. 2d). A similar difference was observed when olfactory bulb volumes were expressed as a proportion of telencephalon volume (p = 0.001, Fig. 2e).

### Comparisons with other taxa

**Figure 3:**
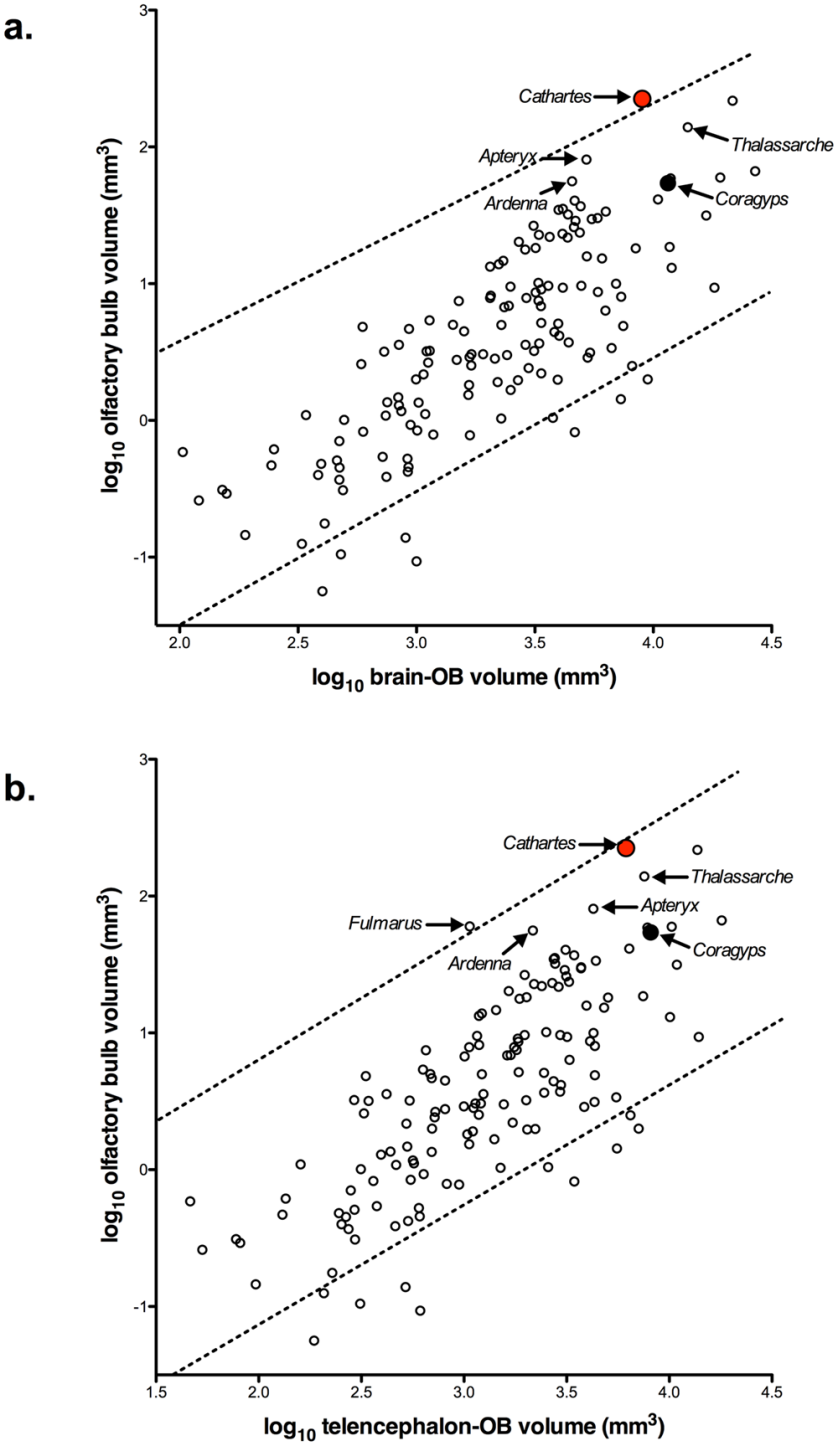
Scatterplots of olfactory bulb (OB) volume plotted against: (**a**) brain minus OB volume; and (**b**) telencephalon minus OB volume. The dotted lines in both scatterplots indicate the 95% prediction interval, which incorporates phylogenetic relatedness. The turkey vulture (*Cathartes aura*) is indicated by the red circle, the black vulture (*Coragyps atratus*) by the black circle and all other bird species by open circles. Arrows indicate the two vulture species as well as several species that have notably keen olfaction (*Apteryx australis*, *Fulmarus glacialis*, *Ardenna tenuirostris* and *Thalassarche melanophrys*).

Olfactory bulb size was positively correlated with both brain and telencephalon volumes across species (Fig. 3, Table 2). Compared with 143 other species, the turkey vulture has significantly larger olfactory bulbs relative to brain volume (Fig. 3a). The turkey vulture even surpasses other species considered to have a highly sensitive sense of smell, such as kiwi and seabirds (*Ardenna tenuirostris*, *Thalassarche melanophrys*). This difference is not as pronounced when olfactory bulb size was scaled relative to telencephalon volume in a bivariate plot (Fig. 3b); the turkey vulture sits just under the upper limits of the 95% prediction interval. Again, kiwi and some seabirds have much smaller olfactory bulbs relative to telencephalon size than the turkey vulture, the only exception being the northern fulmar (*Fulmarus glacialis*).

**Table 2:** Details of the slope and intercept calculated for each allometric relationship (see Figs 3 and 5) with phylogenetic generalized least-squares. λ values and correlation coefficients (r^2^) are also provided. ‘OB’ refers to olfactory bulbs.

Scaling comparison	Slope	Intercept	λ	r^2^
OB volume and Telencephalon	0.8463	−1.8087	0.948	0.4637
OB volume and Brain	0.8999	−2.2001	0.919	0.4607
# Mitral cells and OB volume	0.6756	3.3910	0.268	0.6249
# Mitral cells and Telencephalon	0.4117	2.6818	0.759	0.1122
# Mitral cells and Brain	0.4916	2.2599	0.604	0.1883

**Figure 4:**
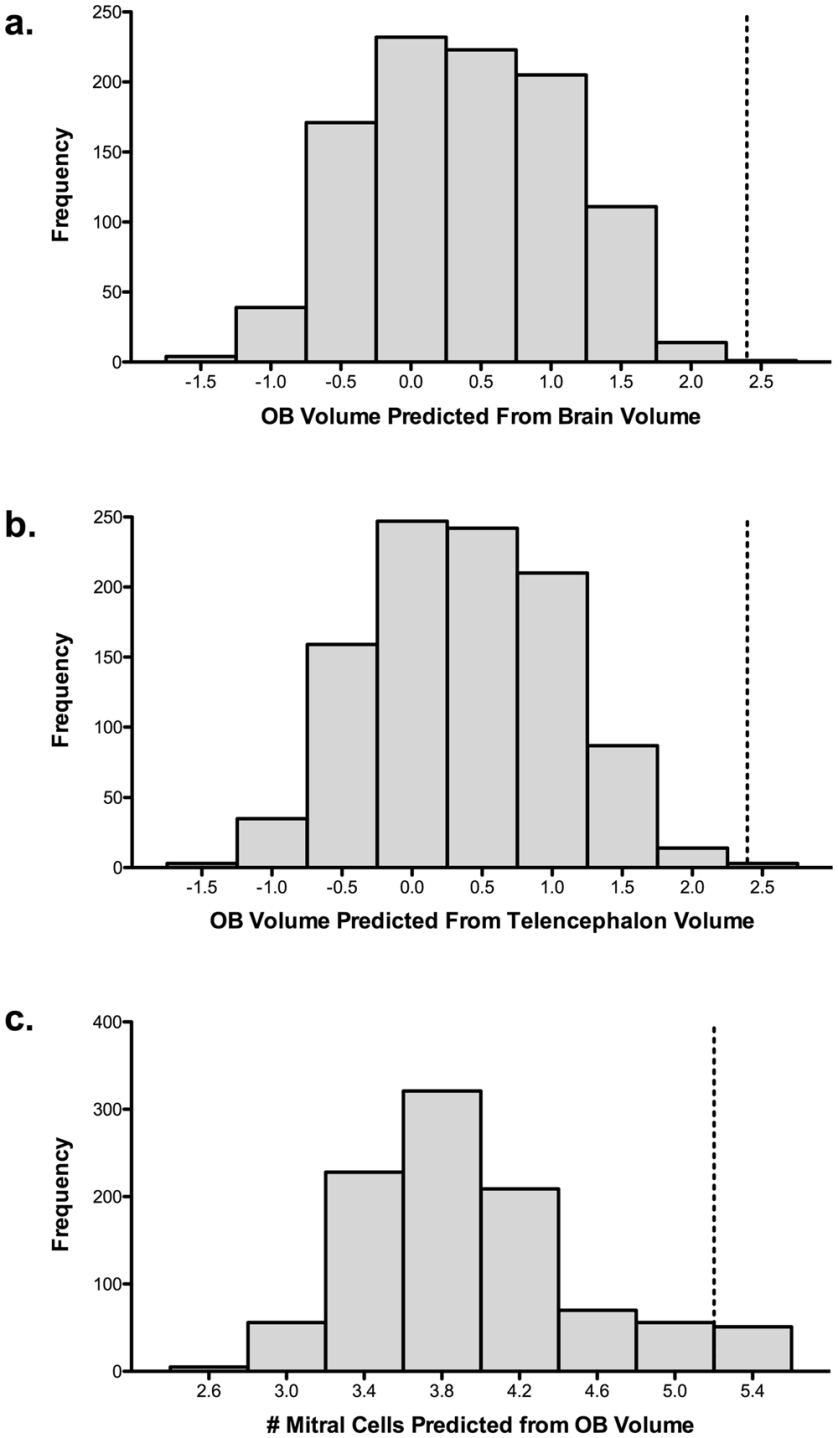
The frequency distribution graphs reflect the posterior probability for the predicted olfactory bulb (OB) volume of the turkey vulture based on phylogeny and the allometric relationship with: (**a**) brain volume; and (**b**) telencephalon volume. In **c**) the frequency distribution reflects the posterior probability for the predicted number of mitral cells of the turkey vulture based on phylogeny and olfactory bulb volume. The observed value of the turkey vulture olfactory bulbs (**a**,**b**) and mitral cells (**c**) is indicated by dotted lines. For the olfactory bulbs, the observed values are beyond the 99^th^ percentiles. The number of mitral cells, however, falls on the 95^th^ percentile.

The posterior probability distributions for predicted olfactory bulb size based on brain and telencephalon volumes corroborate our findings from the scatterplots. Regardless of whether we used brain or telencephalon volume, the observed value for the turkey vulture falls outside of the 95% credible interval (Fig. 4a,b). In other words, the size of the turkey vulture’s olfactory bulbs exceeds what is predicted from allometric relationships with telencephalon and total brain volume.

**Figure 5:**
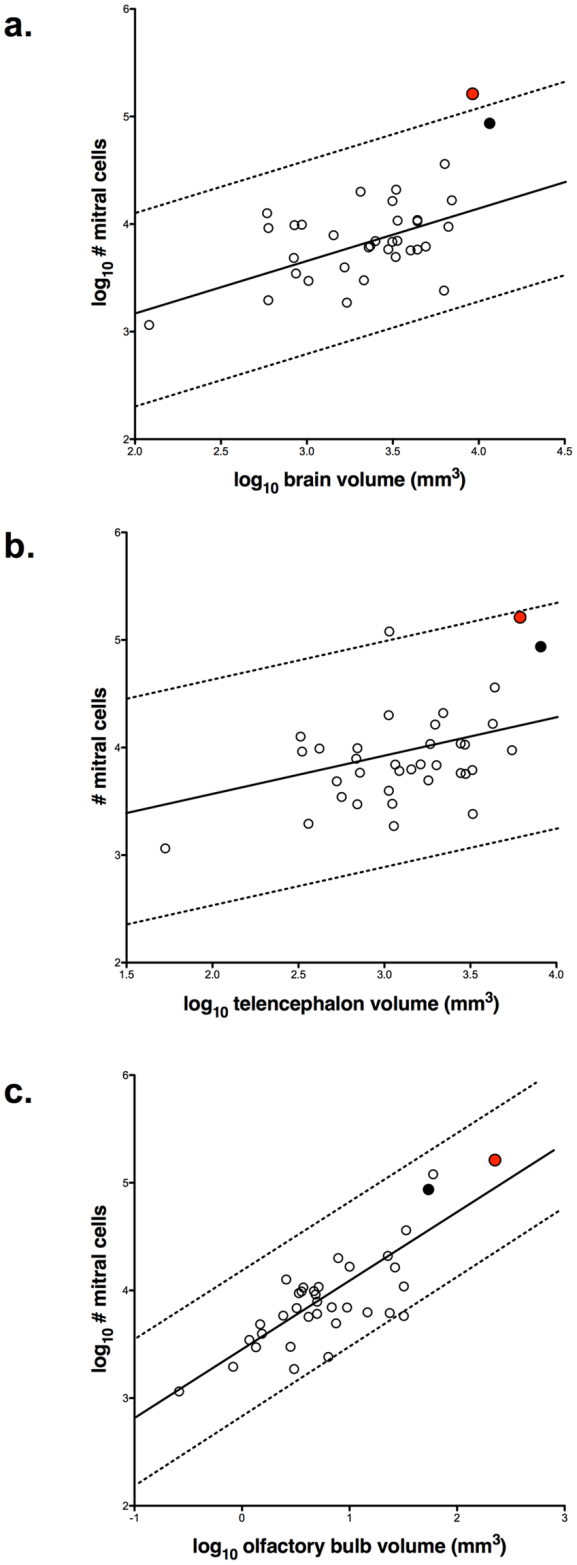
Scatterplots of the number of mitral cells plotted against: (**a**) brain volume; (**b**) telencephalon volume; and (**c**) olfactory bulb volume. As in the previous figures, the turkey vulture is represented by the red circle, the black vulture by the black circle and all other species by the open circles. The solid line in each plot is the least-squares linear regression line and the dotted lines indicate the 95% prediction intervals, which incorporates phylogenetic relatedness.

The number of mitral cells scaled allometrically with brain, telencephalon and olfactory bulb volumes (Fig. 5, Table 2). Although all three of these regression lines were statistically significant, the relationship between mitral cell numbers and olfactory bulb volume was stronger than that between mitral cell numbers and brain or telencephalon volumes (Table 2). In absolute numbers, the turkey vulture has more mitral cells than any other species measured, and it fell narrowly outside the 95% confidence limit in the comparison of mitral cell numbers and brain size (Fig. 5a). Relative to telencephalon size (Fig. 5b) and olfactory bulb size (Fig. 5c), the turkey vulture has the number of mitral cells expected by allometry. The only other comparable outlier in the plots of mitral cell numbers was the northern fulmar, which has more mitral cells than predicted by telencephalon volume (Fig. 5b). The fact that neither the northern fulmar nor the turkey vulture has more mitral cells than predicted by olfactory bulb volume means that their location relative to the prediction intervals in Fig. 5a,b reflects olfactory bulb size (Fig. 3) and not an excess number of mitral cells.

In our posterior probability distributions, the observed number of mitral cells in the turkey vulture is at the upper limit of the 95% credible interval based on the size of its olfactory bulbs, but does not exceed it (Fig. 4c). We therefore conclude that the turkey vulture does not have an excess number of mitral cells relative to the size of its olfactory bulbs.

## Discussion

Previous studies suggested that turkey vultures have a very good sense of smell^8,26,32^ and that black vultures do not^8,28^. Our results confirm this difference in sensory ecology based on both the size of the olfactory bulbs and the number of mitral cells. Turkey vultures have larger olfactory bulbs and more mitral cells than the black vulture, regardless of whether brain size is taken into account. The very large difference between these two species is not the result of a regressed olfactory system in the black vulture; its’ olfactory bulbs are similar in size to those of other bird species with similar brain volumes (Fig. 3). Further, there is no anatomical evidence that the black vulture has better vision than turkey vulture (Fig. 2a–c ^33^). Despite the apparent lack of anatomical specializations in its visual system, based on behavioural observations and experiments, the black vulture probably relies on visual cues, uses social information obtained at roosts and searches for feeding aggregations of turkey vultures to locate carrion^28,34,35^.

Based on our data and that of previous studies^8,26,32^, it is clear that turkey vultures have an enlarged olfactory system that enhances their ability to detect volatile odorants emitted from carcasses. *Cathartes* and *Coragyps* diverged in the mid-Miocene^23–25^ when large areas of savanna woodland in North America led to a speciose community of mammalian herbivores and predators^36,37^. This would have resulted in an abundance of carrion, but also competition among scavengers for access to carcasses. Through the enlargement of its olfactory system, the turkey vulture was able to occupy a new sensory niche among vultures that depended on olfaction. Being able to detect the smell of carrion enables them to find and consume carrion hidden under dense forest cover^26,34^, arrive at carcasses prior to other species^34,35^, and to find small carcasses^38^ that may be overlooked by sympatric vulture species. Similar abilities to locate hidden carrion have been reported in the lesser yellow-headed vulture (*Cathartes burrovianus*) and greater yellow-headed vulture (*C*. *melambrotus*)^8,34,39,40^. Thus, an olfactory-guided foraging strategy is common to all *Cathartes* species and in stark contrast to the foraging modes of black and king vultures (*Sarcoramphus papa*) as well as the Andean (*Vultur gryphus*) and California condors (*Gymnogyps californianus*)^8^.

Compared with other birds, the turkey vulture has the largest olfactory bulbs in absolute terms and relative to brain and telencephalon volumes, rivaled only by the northern fulmar (Fig. 3). Although fulmars and vultures have disparate morphologies, ecologies and behaviors, they have convergently evolved a strong reliance on olfaction to forage successfully for patchily distributed and ephemeral food sources. Seabirds, including fulmars, have sensitive olfactory systems that are used in a range of behaviors, but are especially important for foraging^6^. Because the open ocean lacks visual landmarks, aggregations of prey are temporally and spatially variable and nocturnal foraging is often required, these species rely heavily on olfaction to locate prey^6^ and can detect localized patches of prey via olfactory cues alone^11^. Turkey vultures face parallel challenges when foraging in forested regions where carrion is unpredictable, spatially variable, ephemeral, and obscured from view. To solve the challenges of finding food, both turkey vulture and seabirds rely on olfactory guided foraging and have evolved enlarged olfactory bulbss (Fig. 3)^2,3,8^ and nasal cavities^32,41^ in a clear example of convergent neurobehavioral evolution.

Olfactory abilities are dependent not only on the size of the olfactory apparatus, but also the numbers of neurons. The number of mitral cells in the olfactory bulbs is particularly important because they project to other regions of the brain where odor recognition, decision making and other information processing occurs^1,30,31^. Because alternative methods to counting neurons cannot identify mitral cells^42^, our study is the first to quantify mitral cell numbers across a range of bird species. The number of mitral cells is primarily determined by the size of the olfactory bulbs in birds (Fig. 5c, Table 2) and the turkey vulture did not vary significantly from this relationship (Figs 4c and 5). However, as mentioned above the turkey vulture has greatly enlarged nasal sinuses^32^, which likely reflects a large number of olfactory receptors. The convergence of many olfactory receptors onto each mitral cell would potentially confer an increase in olfactory sensitivity^43,44^, corroborating the ability of turkey vultures to locate accurately smaller and hidden sources of carrion^8,26,27,45^. Although the minimum concentration of carrion odorants required for localization have yet to be tested, based on our results and similarities with the northern fulmar we predict that turkey vultures will have olfactory sensitivity similar to that of many seabirds.

Old World vultures typically live in open habitats where they can soar over large areas in search of carcasses^22^ and heavily forested areas of Africa and Asia are virtually devoid of scavenging birds^26^. In the Western Hemisphere, the turkey vulture and the greater yellow-headed vulture are far more common in unfragmented forests than other New World vultures^22^. Their ability to exploit olfactory cues has enabled turkey vultures to colonize closed-canopy habitats and has likely contributed to the them having the most widespread distribution of any vulture species in the world^22^.

## Methods

### Specimens

Black and turkey vultures were obtained during culling operations conducted in Nashville, Tennessee, USA by the United States Department of Agriculture (USDA) Animal and Plant Health Inspection (APHIS) Wildlife Services in February 2012. All trapping and euthanasia procedures adhere to the Guidelines to the Use of Wild Birds in Research^46^, were approved by the Institutional Animal Care and Use Committee of APHIS and were conducted by APHIS under US Fish and Wildlife Service permit #MB018937-0.

Following euthanasia by CO_2_, facial swabs, large intestine samples and eyes were taken for parallel studies^33,47,48^. The heads of the vultures were then immersion-fixed in 4% buffered paraformaldehyde (pH = 7.4) and left in paraformaldehyde until the brains were extracted. Voucher specimens of all birds were deposited in the research collections of the Division of Birds, National Museum of Natural History, Smithsonian Institution, Washington, DC, USA. The brains were extracted from three individuals of each species, weighed, cryoprotected in a 30% sucrose solution and embedded in gelatin. The embedded brains were then serially sectioned in the coronal plane at a thickness of 40 μm with a freezing stage microtome. Sections were collected in phosphate buffered saline (pH = 7.4) with 0.01% sodium azide, every fourth section mounted onto gelatinized microscope slides, stained for Nissl substance using thionin, and coverslipped with Permount.

### Volumetric Measurements

We created virtual slides of the stained vulture brain sections with a 10 × objective on an Olympus VS-120 Slide Scanner. All volumetric measurements of brain regions were made with Olympus VS-ASW software (v. 2.9). Each region of interest was outlined in the series of thionin-stained sections, summed into a measure of total area, and subsequently converted to an estimated volume by multiplying by both the distance between sections and slice thickness (40 μm). We measured the volumes of the telencephalon, olfactory bulbs and three visual brain regions (optic tectum, nucleus rotundus, and entopallium). These three visual regions comprise the tectofugal pathway, the primary visual pathway in most birds^49,50^. Measuring these visual regions allowed us to test if the purported reliance on visual cues in the black vulture^8^ has led to an expansion of the visual system relative to the turkey vulture.

Olfactory bulb size for eight non-vulture species were measured directly from specimens in our brain collection and combined with additional data from 135 species taken from Corfield *et al*.^3^ for comparative analyses (see supplementary material). All non-vulture brains were processed and measured using the same procedures outlined above and described in Corfield *et al*.^3^.

### Mitral Cell Counts

**Figure 6:**
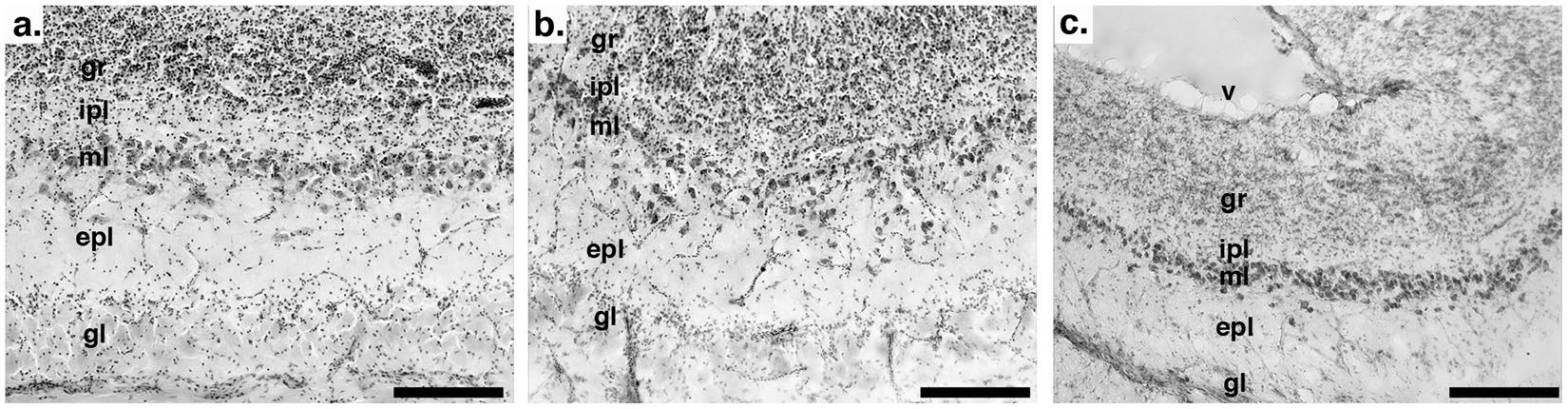
These photomicrographs depict the layers of the olfactory bulbs in: (**a**) turkey vulture (*Cathartes aura*); (**b**) black vulture (*Coragyps atratus*); and **c**) silver gull (*Chroicocephalus novaehollandiae*). The layers are indicated as follows: ‘gl’ – glomerular layer, ‘epl’ – external plexiform layer, ‘ml’ – mitral cell layer, ‘ipl’ internal plexiform layer, ‘gr’ – granule cell layer, and ‘v’ – ventricle. Note that the mitral cells are found primarily within the mitral cell layer in all three species, but some mitral cells are slightly displaced in the external plexiform layer. Scale bars = 200 μm.

We quantified the number of mitral cells for each of the six vulture specimens as well as 34 specimens representing 32 species of 10 different avian orders (see supplementary material), supplemented with data for two species from the literature^51^. Mitral cells were counted using a 40× oil immersion objective (numerical aperture = 1.4) on a Zeiss Imager M2 microscope using the optical fractionator method, as implemented in Stereo Investigator software (MBF Bioscience, Williston, VT, USA). The optical fractionator counts the number of cells in subsamples (counting frames) that are randomly and systematically distributed throughout a region of interest and then calculates total cell populations based on the number of cells across subsamples, density of subsampling, and the dimensions of the counting frame^52^. For the vultures, mitral cells were counted in every 10^th^ section with a frame size of 70 × 70 μm and grid sizes of 275 × 275 μm for the black vultures and 250 × 250 μm for the turkey vultures. Coefficients of error for mitral cell counts varied from 0.03–0.06 for the vultures. Sampling intervals for the mitral cell counts and grid and frame sizes of the optical fractionator varied among the other species due to differences in olfactory bulb size (see supplementary material). Nearly all of the coefficients of error were less than 0.07, with the exception of some songbirds and Anna’s hummingbird (*Calypte anna*), due to the very small size of their olfactory bulbs. Cell counts were conducted in left and right olfactory bulbs in all species separately and then summed, with the exception of songbirds, which possess a single, fused olfactory bulb^3^. In all species, mitral cells were counted within a frame if they were in the mitral cell layer or adjacent external plexiform layer and their soma was large and either a pyramidal or teardrop shape, which differentiated them from the smaller tufted cells and rounder granule cells. The difference in cytoarchitecture across the layers of the olfactory bulb is shown in Fig. 6 for both vulture species and the silver gull (*Chroicocephalus novaehollandiae*).

### Statistical Analyses

Comparisons between the turkey and black vultures were limited to t-tests for both absolute and relative brain measurements due to relatively small sample sizes. Relative brain region volumes were limited to proportions (i.e., brain region volume/total brain volume) for these two species comparisons. To determine if turkey and black vultures were outliers compared with other birds in terms of relative olfactory bulb size and mitral cell numbers, we employed phylogenetic comparative methods^53^. For these analyses, subsets of 1,000 phylogenetic trees were first downloaded for all species in the olfactory bulb volume (145 species) and mitral cell datasets (36 species) from the Bird Tree Project (http://birdtree.org ^54^) using the Hackett *et al*.^55^ backbone. In the first set of analyses, we constructed a 50% majority rule consensus tree for each of the two datasets in the SumTrees program within the Python (v3.6) package DendroPy (v3.12,^56,57^). Data were log_10_-transformed prior to analysis. Allometric equations were calculated with least squares linear regressions using phylogenetic generalized least squares (PGLS) to account for phylogenetic relatedness^58,59^. PGLS allows the covariance matrix to be modified to accommodate the degree to which trait evolution deviates from Brownian motion, through a measure of phylogenetic correlation, *λ* ^60,61^. Our PGLS analyses and maximum likelihood estimates of *λ* were performed using the *caper* ^62^ package in R^63^. We then used the *gls*.*pi* function in the *evomap* ^64^ package to create 95% prediction intervals that include phylogenetic relatedness across species. In each case, the consensus tree was rescaled using the *λ* parameter calculated in *caper*.

In the second set of analyses, we use a phylogenetic prediction approach to test if the size of the olfactory bulbs and number of mitral cells of the turkey vulture is exceptional among birds following the procedures outlined in Nunn and Zhu^65^. Briefly, this method generates a posterior probability distribution for the expected value of a trait using a Bayesian Markov Chain Monte Carlo approach across a tree (or multiple trees) based on a phylogenetic regression with a predictor variable. Importantly, the target species (turkey vulture) was not included in the regression model. We ran 100,100 iterations with a burning rate of 100 and a thin rate of 100 with either brain or telencephalon size as the predictor variable to generate posterior probability distributions of 1,000 values of the predicted size of the turkey vulture olfactory bulbs. Olfactory bulb volume was used as the predictor variable to create a similar posterior probability distribution for the number of mitral cells. Iterations used a random tree from the same set of 1,000 trees discussed above.

## Electronic supplementary material

Dataset 1
