# Supplementary material for: Anatomical evidence for scent guided foraging in the turkey vulture
Source: Sci Rep. 2017 Dec 12;7:17408. doi: 10.1038/s41598-017-17794-0 (PMC5727128; doi:10.1038/s41598-017-17794-0)
Supplement: Supplementary file 1 — Dataset 1 [file 41598_2017_17794_MOESM1_ESM.doc]

**Anatomical evidence for scent guided foraging in the turkey vulture.**

Nathan P. Grigg^1^, Justin M. Krilow^1^, Cristian Gutierrez-Ibanez^2^, Douglas R. Wylie^2^, Gary R. Graves^3^, Andrew N. Iwaniuk^1^

^1^ Department of Neuroscience, University of Lethbridge, Lethbridge, AB, Canada.

^2^ Neuroscience and Mental Health Institute, University of Alberta, Edmonton, AB, Canada.

^3^ Department of Vertebrate Zoology, National Museum of Natural History, Smithsonian Institution, Washington, DC, USA.

**Table 1.** A list of all of the non-vulture species included in our comparative analyses, including sample sizes and volumes of the olfactory bulbs (OB), telencephalon and brain.

| **Order** | **Species** | **Common name** | **n** | **OB volume (mm^3^)** | **Telencephalon volume (mm^3^)** | **Brain volume (mm^3^)** |
| --- | --- | --- | --- | --- | --- | --- |
| Accipitriformes | *Buteo swainsoni* | Swainson's Hawk | 1 | 10 | 4269.59 | 6972.66 |
| Accipitriformes | *Haliaeetus leucogaster* | White-bellied Sea Eagle | 1 | 18.57 | 7456.09 | 11749.03 |
| Anseriformes | *Anas castanea* | Chestnut Teal | 1 | 21.72 | 2889.19 | 4366.8 |
| Anseriformes | *Anas platyrhynchos* | Mallard | 4 | 33.64 | 4386.68 | 6338.32 |
| Anseriformes | *Anas superciliosa* | Australian Black Duck | 1 | 36.87 | 3442.08 | 4973.94 |
| Anseriformes | *Anser anser* | Greylag Goose | 8 | 58.82 | 7828.35 | 11893.22 |
| Anseriformes | *Aythya affinis* | Lesser Scaup | 3 | 28.84 | 3095.14 | 4724.65 |
| Anseriformes | *Aythya americana* | Redhead | 2 | 29.68 | 3717.71 | 5525.77 |
| Anseriformes | *Aythya collaris* | Ring-necked Duck | 1 | 25.92 | 3149.71 | 4652.51 |
| Anseriformes | *Bucephala albeola* | Bufflehead | 2 | 21.96 | 2393.44 | 3673.79 |
| Anseriformes | *Bucephala clangula* | Common Goldeneye | 2 | 30.19 | 3718.39 | 5851.35 |
| Anseriformes | *Chenonetta jubata* | Australian Wood Duck | 1 | 40.54 | 3127.32 | 4697.88 |
| Anseriformes | *Mareca americana* | American Wigeon | 1 | 32.05 | 2778.96 | 4397.88 |
| Anseriformes | *Mareca strepera* | Gadwall | 1 | 18.22 | 2017.22 | 3204.18 |
| Anseriformes | *Mergus serrator* | Red-breasted Merganser | 2 | 23.65 | 3244.79 | 4915.15 |
| Anseriformes | *Oxyura jamaicensis* | Ruddy Duck | 1 | 26.5 | 1973.44 | 3147.21 |
| Anseriformes | *Spatula clypeata* | Northern Shoveler | 2 | 22.74 | 2195.76 | 3310.09 |
| Anseriformes | *Spatula discors* | Blue-winged Teal | 1 | 17.76 | 1865.93 | 2895.75 |
| Anseriformes | *Tadorna variegata* | Paradise shelduck | 3 | 23.18 | 2689.6 | 4157.38 |
| Apodiformes | *Amazilia tzacatl* | Rufous-tailed Hermit | 1 | 0.29 | 81.26 | 158.01 |
| Apodiformes | *Apus apus* | Common Swift | 1 | 1.36 | 438.14 | 754.25 |
| Apodiformes | *Archilochus colubris* | Ruby-Throated Hummingbird | 1 | 0.61 | 135.28 | 250.99 |
| Apodiformes | *Calypte anna* | Anna's Hummingbird | 1 | 0.26 | 53.18 | 120.84 |
| Apodiformes | *Chaetura pelagica* | Chimney Swift | 1 | 1.09 | 159.92 | 342.66 |
| Apodiformes | *Collocalia troglodytes* | Pygmy Swiftlet | 1 | 0.59 | 46.4 | 103.68 |
| Apodiformes | *Phaethornis superciliosus* | Long-tailed Hermit | 1 | 0.15 | 96.7 | 189.25 |
| Apodiformes | *Selasphorus rufus* | Rufous Hummingbird | 1 | 0.31 | 77.52 | 151.64 |
| Caprimulgiformes | *Caprimulgus vociferus* | Caprimulgus sp. | 1 | 3.19 | 342.75 | 733.59 |
| Caprimulgiformes | *Eurostopodus argus* | Spotted Nightjar | 1 | 3.23 | 291.58 | 1141.36 |
| Charadriiformes | *Charadrius vociferus* | Killdeer | 1 | 2.17 | 523.69 | 1073.36 |
| Charadriiformes | *Chroicocephalus novaehollandiae* | Silver Gull | 1 | 2.42 | 720.01 | 2975.65 |
| Charadriiformes | *Chroicocephalus philadelphia* | Bonaparte's Gull | 1 | 9.5 | 1157.11 | 2502.5 |
| Charadriiformes | *Haematopus finschi* | South Island Oystercatcher | 2 | 7.87 | 1757.44 | 2917.7 |
| Charadriiformes | *Himantopus himantopus* | Black-winged Stilt | 1 | 2.91 | 998.82 | 1678.49 |
| Charadriiformes | *Limnodromus griseus* | Short-billed Dowitcher | 1 | 2.65 | 725.11 | 1123.55 |
| Charadriiformes | *Limosa lapponica* | Bar tailed Godwit | 2 | 3.01 | 1563.49 | 2417.27 |
| Charadriiformes | *Scolopax rusticola* | Eurasian woodcock | 2 | 14.71 | 1433.52 | 2338.6 |
| Charadriiformes | *Sterna hirundo* | Common Tern | 1 | 4.49 | 808.53 | 1592.66 |
| Charadriiformes | *Vanellus chilensis* | Southern Lapwing | 1 | 6.89 | 1686.79 | 2461.39 |
| Charadriiformes | *Vanellus miles* | Masked Lapwing | 3 | 8.17 | 1185.57 | 2067.13 |
| Columbiformes | *Columba leucomela* | White-headed Pigeon | 1 | 6.74 | 1006.12 | 2355.21 |
| Columbiformes | *Columba livia* | Pigeon | 10 | 7.86 | 1059.84 | 2055.08 |
| Columbiformes | *Geopelia humeralis* | Bar-shouldered Dove | 1 | 3.2 | 543.29 | 1106.18 |
| Columbiformes | *Phaps elegans* | Brush Bronzewing | 1 | 7.46 | 652.25 | 1517.37 |
| Columbiformes | *Stigmatopelia chinensis* | Spotted Dove | 1 | 5.01 | 686.13 | 1430.5 |
| Columbiformes | *Streptopelia risoria* | Barbary Dove | 1 | 5.4 | 630.98 | 1140.93 |
| Coraciiformes | *Dacelo novaeguineae* | Laughing Kookaburra | 1 | 5.11 | 2451.75 | 3970.08 |
| Falconiformes | *Falco columbarius* | Merlin | 1 | 5.16 | 1847.61 | 3371.43 |
| Galliformes | *Alectoris chukar* | Chukar Partridge | 1 | 1.67 | 1406.39 | 2500 |
| Galliformes | *Bonasa umbellus* | Ruffed Grouse | 3 | 4.99 | 1221.32 | 2288.12 |
| Galliformes | *Callipepla californica* | California Quail | 1 | 0.84 | 549.86 | 1007.84 |
| Galliformes | *Chrysolophus pictus* | Golden Pheasant | 1 | 2.21 | 1726.01 | 3368.73 |
| Galliformes | *Colinus virginianus* | Northern Bobwhite | 1 | 1.11 | 569.85 | 1090.73 |
| Galliformes | *Coturnix coturnix* | Common Quail | 11 | 1.29 | 394.25 | 844.35 |
| Galliformes | *Coturnix japonica* | Japanese Quail | 2 | 3.58 | 419.09 | 846.35 |
| Galliformes | *Dendragapus canadensis* | Spruce Grouse | 3 | 2.83 | 1111.4 | 2146.62 |
| Galliformes | *Gallus gallus* | Chicken | 1 | 3.58 | 1242.46 | 2889 |
| Galliformes | *Meleagris gallopavo* | Turkey | 7 | 6.36 | 3269.02 | 6284.93 |
| Galliformes | *Numida meleagris* | Helmeted Guineafowl | 1 | 1.99 | 2223.28 | 3950.77 |
| Galliformes | *Ortalis canicollis* | Chaco Chachalaca | 1 | 9.1 | 1829.65 | 3373.55 |
| Galliformes | *Pavo cristatus* | Indian peafowl | 5 | 9.65 | 2945.92 | 4977.51 |
| Galliformes | *Perdix perdix* | Grey Partridge | 14 | 0.78 | 946.07 | 1682.55 |
| Galliformes | *Phasianus colchicus* | Ring-necked Pheasant | 14 | 7.5 | 1803.12 | 3286.44 |
| Galliformes | *Tympanuchus phasianellus* | Sharp-tailed Grouse | 1 | 1.9 | 1103.2 | 2204.91 |
| Gruiformes | *Fulica armillata* | Red-gartered Coot | 1 | 34.61 | 2738.46 | 4015.45 |
| Gruiformes | *Gallinula tenebrosa* | Dusky Moorhen | 1 | 20.24 | 1652.54 | 2726.54 |
| Gruiformes | *Porphyrio melanotus* | Pukeko | 3 | 35.22 | 2771.03 | 4186.05 |
| Otidiformes | *Ardeotis australis* | Australian Bustard | 1 | 41.36 | 6377.92 | 10500.97 |
| Passeriformes | *Acanthorhynchus tenuirostris* | Eastern Spinebill | 1 | 0.31 | 294.4 | 489.38 |
| Passeriformes | *Agelaius phoeniceus* | Red-winged Blackbird | 1 | 0.93 | 635.95 | 945.36 |
| Passeriformes | *Baeolophus bicolor* | Tifted Titmouse | 2 | 0.42 | 534.22 | 919.53 |
| Passeriformes | *Carduelis tristis* | American Goldfinch | 1 | 0.4 | 253.08 | 384.58 |
| Passeriformes | *Carpodacus mexicanus* | House Finch | 2 | 0.39 | 462.58 | 747.28 |
| Passeriformes | *Corvus corone* | Carrion Crow | 12 | 2 | 7093.26 | 9477.88 |
| Passeriformes | *Corvus moneduloides* | New Caledonian Crow | 5 | 1.43 | 5558.55 | 7295.4 |
| Passeriformes | *Cracticus tibicen* | Australian Magpie | 4 | 0.82 | 3443.98 | 4664.24 |
| Passeriformes | *Dumetella carolinensis* | Gray Catbird | 1 | 1.48 | 528.68 | 839.35 |
| Passeriformes | *Erythrura gouldiae* | Gouldian Finch | 1 | 0.37 | 273.74 | 472.97 |
| Passeriformes | *Euphagus carolinus* | Rusty Blackbird | 1 | 1.53 | 1060.44 | 1656.56 |
| Passeriformes | *Garrulus glandarius* | Eurasian Jay | 5 | 1.05 | 2571 | 3770.82 |
| Passeriformes | *Grallina cyanoleuca* | Magpie-lark | 1 | 1.82 | 1038.98 | 1668.92 |
| Passeriformes | *Junco hyemalis* | Dark-eye Junco | 3 | 0.83 | 361.77 | 596.06 |
| Passeriformes | *Lichenostomus penicillatus* | White-plumed Honeyeater | 1 | 0.53 | 602.91 | 916.99 |
| Passeriformes | *Manorina melanocephala* | Noisy Miner | 1 | 1.03 | 1505.49 | 2278.96 |
| Passeriformes | *Melospiza melodia* | Song Sparrow | 3 | 1.09 | 467.26 | 742.86 |
| Passeriformes | *Molothrus ater* | Brown-headed Cowbird | 1 | 1.35 | 696.14 | 1021.89 |
| Passeriformes | *Pardalotus punctatus* | Spotted Pardalote | 1 | 0.06 | 186.34 | 400.58 |
| Passeriformes | *Parus carolinensis* | Carolina Chickadee | 1 | 0.1 | 312.22 | 479.65 |
| Passeriformes | *Passer domesticus* | House Sparrow | 9 | 0.46 | 608.6 | 926.38 |
| Passeriformes | *Passerina cyanea* | Inigo Bunting | 1 | 1.01 | 314.79 | 495.68 |
| Passeriformes | *Petroica multicolor* | Pacific Robin | 1 | 0.45 | 266.07 | 473.94 |
| Passeriformes | *Pyrrhula pyrrhula* | Eurasian Bullfinch | 2 | 0.14 | 519.09 | 899.61 |
| Passeriformes | *Sitta carolinensis* | White-breasted Nuthatch | 1 | 0.09 | 612.77 | 1000 |
| Passeriformes | *Spizella passerina* | Field Sparrow | 4 | 0.71 | 280.6 | 472.78 |
| Passeriformes | *Spizella pusilla* | Chipping Sparrow | 2 | 0.48 | 245.79 | 396.8 |
| Passeriformes | *Stagonopleura guttata* | Diamond Firetail | 1 | 0.54 | 375.81 | 720.08 |
| Passeriformes | *Taeniopygia bichenovii* | Double-barred Finch | 1 | 0.18 | 228.4 | 409.27 |
| Passeriformes | *Taeniopygia guttata* | Zebra Finch | 1 | 0.13 | 207.83 | 328.19 |
| Passeriformes | *Troglodytes aedon* | House Wren | 3 | 0.51 | 292.89 | 462.13 |
| Passeriformes | *Turdus merula* | Common Blackbird | 1 | 3.05 | 1208.87 | 1914.09 |
| Passeriformes | *Turdus migratorius* | American Robin | 1 | 3.05 | 1135.32 | 1709.38 |
| Passeriformes | *Zonotrichia albicollis* | White-throated Sparrow | 1 | 1.17 | 563.8 | 864.35 |
| Pelecaniformes | *Ardea cinerea* | Grey Heron | 1 | 18.11 | 5028.04 | 8445.95 |
| Pelecaniformes | *Egretta thula* | Snowy Egret | 1 | 9.64 | 1973.35 | 3612.26 |
| Pelecaniformes | *Nycticorax caledonicus* | Nankeen Night Heron | 1 | 6.84 | 1625.14 | 3360.04 |
| Piciformes | *Indicator minor* | Lesser Honeyguide | 3 | 2.58 | 325.51 | 587.33 |
| Piciformes | *Indicator variegatus* | Scaly-throated Honeyguide | 1 | 4.84 | 332.2 | 597.53 |
| Piciformes | *Melanerpes carolinus* | Red-bellied Woodpecker | 2 | 9.36 | 3180.4 | 4160 |
| Piciformes | *Melanerpes erythrocephalus* | Red-headed Woodpecker | 3 | 10.14 | 2515.73 | 3280 |
| Piciformes | *Picoides pubescens* | Downy Woodpecker | 1 | 2 | 698.83 | 997.53 |
| Piciformes | *Picoides villosus* | Hairy Woodpecker | 1 | 15.84 | 3947.1 | 5250 |
| Piciformes | *Pogoniulus bilineatus* | Yellow-rumped Tinkerbird | 1 | 0.47 | 130.67 | 244.28 |
| Piciformes | *Sphyrapicus varius* | Yellow-bellied Sapsucker | 1 | 4.66 | 696.91 | 934.57 |
| Podicipediformes | *Rollandia rolland* | White-tufted Grebe | 1 | 13.34 | 1183.89 | 2059.17 |
| Procellariiformes | *Ardenna tenuirostris* | Short-tailed Shearwater | 1 | 56.09 | 2164.22 | 4592.4 |
| Procellariiformes | *Fulmarus glacialis* | Northern Fulmar | 1 | 60 | 1067.1 | — |
| Procellariiformes | *Thalassarche melanophrys* | Black-browed Albatross | 1 | 139.26 | 7552.96 | 14129.34 |
| Psittaciformes | *Alisterus scapularis* | Australian King Parrot | 1 | 3.72 | 2938.63 | 4394 |
| Psittaciformes | *Cacatua galerita* | Sulphur-crested Cockatoo | 1 | 2.5 | 6474.04 | 8142.1 |
| Psittaciformes | *Eolophus roseicapilla* | Galah | 1 | 3.38 | 5520.9 | 6666.15 |
| Psittaciformes | *Melopsittacus undulatus* | Budgerigar | 1 | 0.79 | 825.12 | 1177.61 |
| Psittaciformes | *Myiopsitta monachus* | Monk Parakeet | 1 | 4.44 | 2733.13 | 3830 |
| Psittaciformes | *Pionus menstruus* | Blue-headed Parrot | 1 | 2.88 | 3851.82 | 5282.82 |
| Psittaciformes | *Platycercus elegans* | Crimson Rosella | 1 | 4.16 | 2966.4 | 4013.28 |
| Psittaciformes | *Platycercus eximius* | Eastern Rosella | 4 | 1.97 | 2032.15 | 2685.74 |
| Psittaciformes | *Psephotus haematonotus* | Red-rumped Parrot | 1 | 2.52 | 1181.44 | 1705.84 |
| Psittaciformes | *Strigops habroptila* | Kakapo | 1 | 13.07 | 10075.42 | 11979.93 |
| Psittaciformes | *Thectocercus acuticaudata* | Blue-crowned Parakeet | 1 | 3.13 | 4325.91 | 5410 |
| Psittaciformes | *Trichoglossus haematodus* | Rainbow Lorikeet | 1 | 3.66 | 2459.34 | 3301.16 |
| Sphenisciformes | *Eudyptula minor* | Little Penguin | 1 | 4.9 | 4338.36 | 7473.94 |
| Sphenisciformes | *Spheniscus magellanicus* | Magellanic Penguin | 1 | 31.48 | 10890.21 | 16756.76 |
| Strigiformes | *Aegolius acadicus* | Northern Saw-whet owl | 1 | 3.22 | 2009.9 | 3142.86 |
| Strigiformes | *Athene cunicularia* | Burrowing Owl | 1 | 15.28 | 4813.8 | 6090 |
| Strigiformes | *Bubo scandiaca* | Snowy Owl | 1 | 9.35 | 13921.62 | 18127.41 |
| Strigiformes | *Tyto alba* | Barn Owl | 1 | 8.71 | 4108.76 | 5849.81 |
| Struthioniformes | *Apteryx mantelli* | North Island Brown Kiwi | 2 | 80.76 | 4267.72 | 5298.95 |
| Struthioniformes | *Dromaius novaehollandiae* | Emu | 1 | 217.63 | 13695.99 | 21829.88 |
| Struthioniformes | *Rhea americana* | Rhea | 1 | 59.67 | 10281.31 | 19227.8 |
| Struthioniformes | *Struthio camelus* | Ostrich | 1 | 66.52 | 17984.78 | 27006.26 |
| Suliformes | *Phalacrocorax auritus* | Double-crested Cormorant | 1 | 8.03 | 4341.73 | 7323.36 |
| Tinamiformes | *Nothura darwinii* | Darwin's Nothura | 1 | 2.77 | 809.09 | 1482.37 |
| Tinamiformes | *Rhynchotus rufescens* | Red-winged Tinamou | 2 | 8.63 | 1838.21 | 3195.65 |
| Tinamiformes | *Tinamus major* | Great Tinamou | 1 | 13.87 | 1221.88 | 2242.13 |

**Table 2.** Stereological parameters, coefficients of error (CE) and mitral cell counts for all of the non-vulture species measured. The number of mitral cells is rounded to the nearest whole number for each species.

| **Order** | **Common name** | **Species name** | **n** | **Frame size (μm)** | **Grid size (μm)** | **CE** | **# mitral cells** |
| --- | --- | --- | --- | --- | --- | --- | --- |
| Accipitriformes | Swainson’s Hawk | *Buteo swainsonii* | 1 | 50 x 50 | 150 x 150 | 0.06 | 16,639 |
| Anseriformes | Mallard | *Anas platyrhynchos* | 1 | 50 x 50 | 150 x 150 | 0.03 | 36,232 |
|  | American Wigeon | *Mareca americana* | 1 | 50 x 50 | 150 x 150 | 0.04 | 10,905 |
|  | Gadwall | *Mareca strepera* | 1 | 60 x 60 | 225 x 225 | 0.05 | 5,796 |
|  | Red-breasted Merganser | *Mergus serrator* | 1 | 50 x 50 | 150 x 150 | 0.05 | 6,173 |
|  | Ruddy Duck | *Oxyura jamaicensis* | 1 | 50 x 50 | 150 x 150 | 0.04 | 16,375 |
|  | Northern Shoveler | *Spatula clypeata* | 1 | 50 x 50 | 150 x 150 | 0.03 | 20,905 |
| Apodiformes | Anna's Hummingbird | *Calypte anna* | 1 | 50 x 50 | 150 x 150 | 0.19 | 1,154 |
| Charadriiformes | Silver Gull | *Chroicocephalus novaehollandiae* | 1 | 60 x 60 | 225 x 225 | 0.04 | 5,830 |
|  | Bonaparte's Gull | *Chroicocephalus philadelphia* | 1 | 50 x 50 | 150 x 150 | 0.05 | 6,945 |
|  | Eurasian Woodcock | *Scolopax rusticola* | 1 | 60 x 60 | 225 x 225 | 0.04 | 6,263 |
| Columbiformes | Pigeon | *Columba livia* | 1 |  |  |  | 20,000* |
|  | Spotted Dove | *Spilopelia chinensis* | 1 | 50 x 50 | 150 x 150 | 0.06 | 7,873 |
| Falconiformes | Merlin | *Falco columbarius* | 1 | 50 x 50 | 150 x 150 | 0.04 | 10,781 |
| Galliformes | Ruffed Grouse | *Bonasa umbellus* | 2 | 50 x 50 | 150 x 150 | 0.05 | 6,068 |
| Galliformes | Japanese Quail | *Coturnix japonica* | 1 | 50 x 50 | 150 x 150 | 0.06 | 9,783 |
|  | Spruce Grouse | *Falcipennis canadensis* | 1 | 50 X 50 | 150 X 150 | 0.07 | 3,002 |
|  | Turkey | *Meleagris gallopavo* | 1 | 50 x 50 | 150 x 150 | 0.07 | 2,409 |
|  | Ring-Necked Pheasant | *Phasianus colchicus* | 1 | 50 x 50 | 150 x 150 | 0.07 | 4,950 |
| Passeriformes | Grey Catbird | *Dumetella carolinensis* | 1 | 50 x 50 | 150 x 150 | 0.05 | 4,851 |
|  | Rusty Blackbird | *Euphagus carolinus* | 1 | 50 x 50 | 150 x 150 | 0.07 | 3,962 |
|  | Dark-Eyed Junco | *Junco hyemalis* | 1 | 50 x 50 | 150 x 150 | 0.07 | 1,958 |
|  | Brown-headed Cowbird | *Molothrus ater* | 1 | 50 x 50 | 150 x 150 | 0.07 | 2,965 |
|  | American Robin | *Turdus migratorius* | 1 | 50 x 50 | 150 x 150 | 0.08 | 1,863 |
| Passeriformes | White-throated Sparrow | *Zonotrichia albicollis* | 2 | 50 x 50 | 150 x 150 | 0.06 | 3,474 |
| Pelecaniformes | Nankeen Night Heron | *Nycticorax caledonicus* | 1 | 60 x 60 | 225 x 225 | 0.04 | 6,988 |
| Piciformes | Lesser Honeyguide | *Indicator minor* | 1 | 50 x 50 | 150 x 150 | 0.04 | 12,637 |
|  | Scaly-Throated Honeyguide | *Indicator variegatus* | 1 | 50 x 50 | 150 x 150 | 0.05 | 9,186 |
|  | Yellow-Bellied Sapsucker | *Sphyrapicus varius* | 1 | 50 x 50 | 150 x 150 | 0.07 | 9,868 |
| Procellariiformes | Northern Fulmar | *Fulmarus glacialis* |  |  |  |  | 120,000* |
| Psittaciformes | Australian King Parrot | *Alisterus scapularis* | 1 | 50 x 50 | 150 x 150 | 0.06 | 10,644 |
|  | Galah | *Eolophus roseicapilla* | 1 | 50 x 50 | 150 x 150 | 0.07 | 9,449 |
| Psittaciformes | Crimson Rosella | *Platycercus elegans* | 1 | 50 x 50 | 150 x 150 | 0.06 | 5,688 |
| Strigiformes | Northern Saw-Whet Owl | *Aegolius acadicus* | 1 | 50 x 50 | 150 x 150 | 0.07 | 6,853 |

* data from Wenzel & Meisami (1987)
